# On the Function of the Minute Arteries

**Published:** 1866-08

**Authors:** J. S. Jewell

**Affiliations:** Prof. of Anatomy in Chicago Medical College


THE
### CHICAGO MEDICAL EXAMINER.

N. S. DAVIS, M.D., EDITOR.

VOL. VII. AUGUST, 1866. NO. 8.

### Original Contributions.

### ARTICLE XXV.

### ON THE FUNCTION OF THE MINUTE ARTERIES.

BY J. S. JEWELL, M.D., Prof. of Anatomy in Chicago Medical College.

I desire, in the present article, to draw attention to certain facts and views, and some of the consequences to which they seem to lead; which, if they are not new, are not practically recognized as having the importance they seem to me to have.

The points may be briefly stated in the form of propositions, each of which will be in some measure discussed, and, subsequently, their practical consequences considered. They are as follows:—

I. The small arteries or arterioles, previous to their termination in the capillaries, are purely muscular in their middle coat—*contractile* and not *elastic*, as the large arteries are.

II. These small arteries are supplied by, and are as truly under the control of, the organic nervous system as, for example, the esophagus is or small intestines, etc.

III. That it is the special office of these muscular vessels to impart a new momentum to the blood necessary to circulate it through the capillary system, as truly as it is the office of the heart to impart the initial movement to the blood, or of the ejaculatory ducts, esophagus, small intestines, and the like to circulate their contents.

1. That the arteries, even of the larger kind, are provided with muscular tissue in their middle coat, is well known and easily demonstrated. That the muscular tissue comparatively increases, as we proceed towards the distal end of the arteries, is a well-known fact. That it is of the unstriped or involuntary kind is also well known; and, that while the larger arteries have the thick middle coat, mainly constituted of fibrous tissue, largely of the elastic kind, with some unstriped muscular tissue imperfectly developed, and, therefore, probably imperfectly contractile, that, on the other hand, the smaller and finer arteries, previous to their termination in the capillaries, have the elastic tissue found in the middle coat elsewhere, *entirely* replaced by muscular tissue of the involuntary kind, while the capillaries are entirely devoid of either fibrous or muscular tissues, and present only a thin, almost structureless wall, apparently just sufficient to separate the blood within from the tissue without the vessel. These facts have been abundantly demonstrated by direct evidence. But they can also, in quite an interesting manner, be established by indirect evidence which I deem it needless to introduce, more especially, since some of it will be introduced in another part of this paper, for a somewhat different purpose.

There can be no doubt then, as to the muscularity of the middle coat of the small arteries, neither can there be any doubt as to the elasticity of the larger arteries. Considering, for sake of convenience, the arterial tubes as one tube throughout their course, we have a vessel terminated at either end by a muscular structure—at one end the heart, at the other the *distal* end of the artery, the muscular structure already referred to, both contractile; while the intervening tube, may, practically speaking, be called *elastic* and *not* contractile. The heart is richly supplied with nerves from the system of organic life, and even has a large number of ganglia imbedded in its structure. In turning to the small muscular arteries, to ascertain if *they* are also supplied with the nerves of organic life, we are brought to our second proposition.

2. That the power and influence of the organic nervous system in the various processes of organic life, as we witness them in *animal* life, has not been sufficiently attended to, I have no doubt. That it constitutes, taken altogether, the fountain, or centre, or source, so to speak, of the excitant energy or force, rythmically or constantly dispensed to the various parts of the organism, concerned in executing the organic process on which the continuance of life depends, does not seem to have been sufficiently considered in all its relations, generally speaking. That the various organs, as, for example, the esophagus, the stomach, the small and large intestines, execute their movements through the agency of the involuntary muscular tissue with which they abound, and that this muscular tissue is normally made to contract by a direct stimulating influence or power conveyed to it, for which it directly depends on this organic nervous system, is *also* well understood. It is well known that all the normally involuntary movements of the body, with, perhaps, some rare exceptions, are executed by this unstriped muscular tissue, excited to action by or through the organic nervous system, as in the examples already given.

Now, since it is a well-established fact, that the small arteries are purely muscular, to the extent already shown, and since this muscular tissue has been shown to be of the involuntary kind, let us see if these small arteries are also supplied with organic nerves, as other parts are from which they materially differ only in size, and whether it can be shown that these small vessels are excited and controlled as other parts are, by the sympathetic nervous system.

When we consider how thickly woven plexuses occur on most all the arteries, as on most of the larger arteries in the cranial cavity, and as, almost without exception, the arteries given off from the aorta within the thoracia, abdominal and pelvic cavities have; and the fact so often demonstrated, that branches or twigs from this nervous system do actually ramify on the small arteries, at least in some parts of the body, it would seem not to require more than a simple reference to the fact. But it is important, as we will see, that this fact, if it be such, should be fully admitted.

If these vessels are muscular, it would not be unreasonable to expect, *a priori*, they are supplied with nerves, as we know the same kind of muscular tissue elsewhere to be, and we have just seen it can be shown that such is the fact, at least, in many parts of the body. Suppose, then, we admit these muscular vessels *are* thus supplied from the organic nervous system.

This being admitted, it seems scarcely possible to avoid the conclusion that these structures are related here, for the same purpose they are related elsewhere, for, *viz.*:—These nerves are here for the purpose, in part at least, of conveying to this muscular tissue the excitant influence which is the direct cause of its action or contraction. The *inference*, I say, seems almost unavoidable, that such *is* the relation. Now, while it is somewhat difficult to offer direct proof that this is true, yet we are not wholly destitute of such proof. Among others, I will mention one experiment, well known, which seems to me, when attentively considered, to be sufficient to show that these muscular vessels are under the control of this nervous system.

If the sympathetic nerve be divided high up in the neck, on one side, in a rabbit for example, immediately after, we have visible expansion of the small arteries and capillaries, etc., of the conjunctiva and ear of that side and, doubtless, in other parts, as a result. [BERNARD.] The vessels which have suffered the most remarkable expansion in this case, are the small muscular arteries.

Now, in this case, it seems to me, the only way we can explain the appearances, is to suppose we have done for the muscular structure of the affected vessels what we do for a voluntary muscle, by dividing the nerve which leads to it, it is paralyzed, ceases to contract, relaxes, ceases, in great measure, to resist the expansive pressure of the blood, and does what we would *expect* it to do, *dilates*.

This experiment seems to *me* to show, that what is known to be true, *e.g.*, of the esophagus, from which the vessel differs essentially only in size, is *also* true of these muscular tubes or vessels—that, normally, they *do* contract under an influence derived from the organic nervous system—and, that when, for any reason, this excitant influence is withdrawn, they cease to contract, as we would expect from analogy. Indeed, we cannot deny this, without creating the necessity of explaining why we have this relation between contractile and nervous tissues in *one* case, without the consequences universally observed elsewhere. Then let us admit the second proposition, at least as probable, *viz.:*—That these small arteries are supplied by, and are as truly under the control of, the organic nervous system as the esophagus is, or small intestines, etc.

3. In order to prepare the way for fairly considering the third proposition, let us begin by recalling, that it is the office of the heart to communicate the initial impulse to the blood. The force exerted by the heart is expended in two ways:—1st. In setting the blood in motion. 2d. In opposing the elasticity of the arteries, by which latter the force so expended is *conserved.* The arteries, by the means just referred to, are normally maintained in a state of tension. The force conserved by the artery, by virtue of its elasticity, is expanded in reäcting on the contained column of blood, especially in the interva between one ventricular contraction and the next which succeeds it. The blood, being unable to return toward the heart, is steadily forced onward, toward the distal end of the artery, with the exception of the rythmical impulse communicated by the heart, which impulse is felt to the remote extremity of the artery, and to which, in all probability, the pulse is due.

Now, it becomes a question, whether, by reason of friction and other causes, the momentum of the blood is not so much diminished as to make it difficult, if not impossible, to circulate it through the exceedingly small, tortuous capillary passages, unless some means exist for communicating a new impulse to the blood which has just entered, or is about to enter, the capillaries. It certainly is not unreasonable to expect such a means. Now, if the muscular arrangement which we have seen, does actually exist at the distal end of the arteries, and which is provided with a nervous apparatus, is not for some such purpose as this, the question, as to what *is* its use, seems to me unanswerable.

1st. *Analogical Evidence.*—I have before remarked, that these vessels do not differ essentially from some other organs, the action of which is well understood, only in size. You have a tube with unstriped muscular walls, supplied in both cases by the organic nervous system.

Now, we know very well how the esophagus, for example, during the act of swallowing, we will say, of water, is made to propel it in a certain direction, and even against considerable resistance, as that of gravity, or how its action may be reversed, as in eructations and vomiting. I say, that, in this case, a fluid is propelled along a straight tube with considerable force, by its rythmical contraction being rythmically supplied with its contents, as the esophagus is in the successive acts of deglutition. Concerning this, there can be no doubt. In the case of the artery with muscular walls, we have exactly the same conditions—a fluid transmitted through the tube, rythmically in accordance with the heart's action. The question now is, whether the fluid (the blood) passed into the *latter* vessel with an impulse, will, as in the other case, excite contraction. I confess that I cannot see but that it will; and if we deny that it does, we are confronted by the impossible task, as it seems to me, of explaining *why* it does not in the one case, when it *does* take place in the other.

Evidence of the same kind, from the stomach, the intestines, the ejaculatory ducts, and from other members of the animal kingdom, might be given, if I had the time or deemed it necessary.

2d. *Direct Evidence.*—But we are not confined to such evidence as that just adduced. There is positive evidence to show that the vessels under consideration will contract like other muscular organs, under the influence of stimuli. If the mesentery of a frog is drawn out and placed under the microscope, and a galvanic current be transmitted through one of these small muscular vessels, it will immediately contract, until its size is *very* much diminished. But, if the current be too powerful, or too long continued, the vessel ceases to respond to the stimulus, and not only returns to its normal size, but becomes much enlarged, presenting an aneurismal bulging at the place supposed, becoming much more visible than before. [WEBER.]

The same phenomena may be observed, still better, in the bat's wing. In *this* case, under strong stimulation, these muscular vessels become so much reduced in size as to appear almost obliterated. If too powerfully or too long stimulated, the vessels lose their susceptibility to the stimulant, and relax until they finally attain, in some instances, many times their natural size. [PAGET.]

No better evidence of the kind could be required, to show that these are susceptible and do contract, on the one hand, or that they may be paralyzed, on the other. The question once again recurs now—If it is not the office of these vessels to to propel the blood, what *is* their office? Under like circumstances, we see other organs propelling fluids, why not the same happen here?

To change the view a little. It is perfectly reasonable to suppose when a vessel is suddenly diminished in calibre, it will, for this reason, obstruct the flow of blood through it, and that, as a consequence, the fluid will accumulate behind the point of constriction. It is also perfectly reasonable to suppose that if, for any cause, the vessel should *cease* to contract, either because it had lost the power, or because the excitant influence had been withdrawn, no matter which way now, it should become distended with its proper contents as it relaxed, in fact, that there should be an accumulation of the contents of the vessel at such place. Such phenomena as those I have just mentioned, have been actually observed innumerable times, but especially the last mentioned; and I have to add, in this case, that the blood *is invariably retarded in its movement, or even arrested.* One cannot, it seems to me, witness such phenomena and not be inclined to the view, that the vessels referred to really have as their distinct office that of propelling the blood which enters them, onward, toward, or through the passive, tortuous capillaries.

But, again, there is a class of animals destitute of a heart proper; but in which, nevertheless, the blood is propelled exclusively, as it seems, by vessels similar to those we are now considering—vessels possessed of muscular walls, which coneration will show how powerful and efficient, in the circulation of the blood, simple vessels may become. If similar vessels in the *one* case can *circulate* the blood, it is not unreasonable to suppose in the *other* case, they can *help* to do it. The circulation called the portal—the circulation of the blood *from* the placenta to the child—and many other examples might be given, to show how efficient simple vessels may be in the circulation of the blood.

But, while the evidence is not so complete as could be desired, which has been presented, it is, nevertheless, sufficiently so to show that, in all probability, it is one of the principal offices of these muscular vessels, supplied and controlled by the organic nervous system, to impart a new momentum to, or circulate, or assist in circulating, the blood through the capillary passages.

Within the limits of this paper, only a small portion of the evidence can be introduced and discussed, which might be, on which these propositions rest. But enough, it seems to me, *has* been introduced to give the only one of the propositions, concerning which there could arise much doubt, a high degree of probability. I am now engaged, as my occupations will permit, in an experimental investigation of this subject, more particularly in reference to inflammation, and, so far as I may now conclude from my own observations, the above propositions express the truth. Admitting, then, the propositions as probable, I now desire to consider a few of the most prominent objections, which may be or have been made to these views. They are directed mainly against the third proposition. The first two can be supported on positive evidence; the third is, in great measure, a matter of inference.

OBJECTIONS.

I. The objection which would seem to have the most force, is, that after carefully examining these small muscular vessels under the microscope, one cannot perceive such a rythmical action as has been supposed. At first sight, it would seem that if such a contraction occurred it could easily be seen. But this is by no means so certain as one would think. Let any one lay bare the heart of an animal while in action, and undertake to say, from looking at it, what its peculiar action is. It is notorious, that competent observers have come to opposite conclusions as to the heart's action, in many respects, for example, as to whether the heart lengthens when it contracts.

Let anyone lay bare a small artery and watch it, and if they can determine it moves at all, they will find it impossible to determine what the real *character* of the movement is. The movement is not only so slight, but occurs with such quickness the eye cannot certainly follow it, and this is especially true of these minute muscular arteries, in which the movements are somewhat different from what they are in the larger elastic arteries, where we have a distinct pulse. I maintain that the movement must take place much more slowly than it does, and, at the same time, the eye be very accurate and quick if it detect it at all certainly. To my mind, this is no sufficient reason for denying the action of these vessels, as claimed. It certainly cannot be objected, they do not have the capacity for action, and that the circumstances make it unreasonable for us to expect it.

II. But it has been objected, that fluids circulate in plants, and can be raised in capillary tubes, a short distance, without the aid of contractile vessels.

As regards the circulation in plants, it is to be remarked there are numerous differences, when compared with the circulation of animals, to be considered.

The circulation is very slow in plants, when compared with that in animals, and, moreover, plants have tubes with firm, unyielding walls, able to resist atmospheric and other pressure, and are supported by a firm, woody texture, making the circumstances quite different from what we have in animals, in which the vessels are comparatively loose and flaccid, and either elastic or contractile, for most part, and ramify in soft, yielding structures; and as regards the capillaries, they are tortuous, have exceeding fineness, and really oppose *physical* resistance to the circulation of the blood through them. I do not consider the cases as sufficiently parallel to give the argument much force, that, because fluids circulate through plants, apparently without requiring contractile vessels, that the same must therefore necessarily be true of animals. Certainly, the two cases are, in many important respects, so different, as to destroy, in great measure, their parallelism. Then I can see no objection to admitting more than one way of circulating fluids, if the facts require us to, as indeed I think they do. Certainly, such would be more rational than many of the explanations are, which have been offered, of the circulation. But, after all, I am far from denying that the means, whatsoever they may be, by which fluids are circulated in plants, may not also *assist* in the circulation of the blood and fluids in animals.

III. But another explanation has been given of the circulation of the blood through the capillaries, and which *has* even been supposed sufficient to account for the *entire* circulation. It is said, there is an *affinity* between the blood and the tissues, by which the blood is *drawn* or *attracted into* the capillaries. That there is some affinity between the blood and tissues, there can be no question. But when we come to consider whether this affinity is concerned in circulating the blood, we come to quite a different question. Now, I believe this affinity has but little, if anything, *to do* in the circulation of the blood. Nay, I would sooner think it would *embarass* the circulation, than that it aided it. This affinity is, I believe, admitted to be exerted in the capillaries. I understand it to be an *attraction* between the tissue without and the blood within the capillary. I also understand this affinity to be of that intimate kind *only* exerted between contiguous particles, and *not* between particles remote from each other. And, so far as I have any notion of this affinity at all, I understand it to be a force by which contiguous particles are determined or attracted toward each other, modified in some obscure way by the life forces. I do not understand this force to be exerted, as already said, only between contiguous particles, and when it *is* exerted that the *effect* on the *moving* particles (say in the blood) is to *detain* them, rather than drive them forward to make way for their successors. I confess my entire inability to imagine how this power shall act, either on the blood which is subject to it, so as to propel it onward, or on the remote blood advancing from the heart, so as to *attract* or *draw* it into the tissues, without postulating forces or capacities which have only the merest hypothetical existence, and without involving myself in difficulties which are, to say the very least, inextricable and incomprehensible. It is bad enough, when we come to explain the normal circulation, but the difficulties are immeasurably increased, when we come to explain the abnormities of the circulation, as in congestions, inflammations, and the like.

I cannot, within the limits of this article, do more than allude to this objection, to fully analyze which, and to expose its futility, would require of itself a separate article. I am as far as any one from insisting on a physical explanation, only where such an one harmonizes so fully with, and seems to be so imperatively demanded by, the facts.

While I, of course, admit the existence of this affinity, I deny that it has any considerable share in the circulation of the blood, if, indeed, it has any. Other objections *have* been made, or might be, but they seem to me to be destitute of any considerable force. So far, then, as the objections go, they leave our third proposition with, at least, the same degree of probability attaching to it that the evidence gave it.

To sum up, we seem to have reached, with more or less certainty, the following conclusions:—

1. The small arteries are purely muscular in their middle coat.

2. They are supplied by organic nerves.

3. They are under the control of these nerves, and one of the indispensable conditions of the action of these muscular vessels is, that they receive the excitant influence it is one office of these nerves to convey to them.

4. It is the office of these vessels to assist in the circulation of the blood through the capillaries, in performing which, they are probably the principal agents. They do this as other organs analogous to them circulate fluids, examples of which have been given.

While the blood may be conveyed to the small vessels and capillaries by the agency of the heart and *elastic* arteries, there is reason to believe the blood would either be imperfectly, or not at all, circulated through the capillaries into the veins, were it not for this muscular apparatus, by which the blood receives a new momentum, necessary, and sufficient to circulate it through the capillaries, in spite of the difficulties interposed by the latter.

PRACTICAL APPLICATION.

It will only render more probable the truth of the above conclusions, if practical application can be made of them in actual pathological states. I propose, briefly, to make one such application here. One of my chief reasons for presenting this article was, that the application of such views seemed to me to throw new light on the pathology of cholera, which we are constantly expecting in the United States. I had, however, long since began the examination of this subject, in reference to inflammation.

Now let us see if, by keeping these conclusions in view, we can get at a more rational view of the pathology of cholera than has commonly prevailed. And surely, in so fearful a disease as this, concerning which so little is truly known, no serious attempt to throw even a feeble light on its nature should be disregarded.

Admitting, then, that the office of these small muscular arteries is as has been stated, and that one indispensable condition of their action is, that they shall receive rythmically from the organic nervous system the excitant influence on which their action directly depends—I say, *admitting* this—we are in position to see if any agent whatsoever shall so act on the nervous system as to diminish (we will say) its energy, that in proportion as these vessels depend on it in the way indicated, in exactly that proportion will they be affected, or fail of performing their proper office; and, just so far as the circulation depends on these vessels, just so far will it be affected or fail. If, for any reason, the stimulating influence of the organic nervous system should be withdrawn or destroyed, entirely or partially, death will immediately follow, in the one case, or, in the other, more or less marked depression of all the organic functions will follow, which depend on the integrity of the organic nervous system and a constant supply of blood. This will be attested, of course, by prostration, coldness of the surface and body generally, from failure of the "chemico vital" processes on which the production of heat depends—feeble pulse, feeble action of the heart, deficient capillary circulation and the like, but especially the latter.

Now, I must say, admitting the views above insisted on, and supposing (as I think we reasonably may) a cause which shall act as I have indicated, the consequences related are logically deducible. I will now state, briefly, what I believe to be the history of a marked case of cholera, that it may be compared with the views and general statements just made:—

1. I believe cholera to depend on a specific epidemic cause, probably unknown save in its effects.

2. This cause I believe to be introduced into the blood. When there, it may, and probably does, act on the blood; but its most important, if not its primary action, is on the organic nervous system.

3. That the immediate effect of its action on this nervous system is to impair or destroy its energy in a manner more or less marked, and mediately or secondarily all the organic functions which depend on this nervous system.

4. That the destruction or withdrawal of this energy, or excitant power, according to its completeness, in the same degree affects the system of organs whose office it is to supply the various parts of the body with nutriment or blood, namely, the heart and muscular arteries, particularly the latter, which are at the threshold of the tissues, and, as a consequence, we have failure, more or less, of those intimate actions between the blood and tissues, on the proper performance of which, the welfare of the organism depends, and that the symptoms are prostration, paleness of the surface, ghastly appearance of the countenance, coldness, feeble action of the heart and small vessels, feeble pulse, local congestions, collapse, failure of all the true secretions, for want of a healthy supply of blood, and also of that excitant energy on which they depend for successful working, no less truly than do the heart and muscular arteries.

5. That the essential cause of cholera is *not* retained excretory matter, nor, necessarily, a change in the blood *per se*, but, as has been stated above; and that the primary or principal danger in this disease is this profound affection of the organic nervous system, from which the patient may die at once in collapse before discharges occur.

6. But another danger arises secondarily, in most cases, that is from the excessive discharges by which the blood becomes suddenly so much impoverished and so viscid, as to render it alike unfit to maintain life on the one hand, or, on the other, to circulate through the capillaries. These discharges occur in the following manner:—Blood is sent freely and abundantly to the abdominal viscera, or to all those parts, the blood from which is collected by the *portal* vein. The blood once *in* the portal vein, instead of passing as the blood *elsewhere* does, unobstructed into the great blood channels which lead directly to the heart, instead of this, I say, it is required to pass through *another set of capillaries*. Now, if what we have tried to show is true, the small vessels in the intestinal walls (and especially in the soft, yielding mucous membrane) have lost, in greater or less measure, their contractile power, as they have everywhere, against this anomalous condition of the portal circulation there is no help. We have *here* a natural difficulty in the circulation experienced nowhere else in the body save, probably in the kidney.

If our conclusions above given are *true*, it is easy to see why the blood is *detained* here as nowhere else. It is also easy to see, since there is no interruption to the *supply* of blood to the abdominal viscera, there must speedily be a great *accumulation* of blood in the vessels which ramify in the mucous membrane (we will say) of the stomach, and bowels, and in the portal vessels. It is now easy to see, if this state shall continue, that the time will soon come when the vessels will inevitably relieve themselves by copious watery exudation or effusion *on the mucous*

*membrane* of the stomach and bowels, and that this membrane thus *congested* will be somewhat irritable, to say the least, and that there will be vomiting and purging of a watery fluid, which is nothing more nor less than the serum of the blood, and that it will occur to an extent which will depend on a variety of circumstances. The patient rapidly loses the watery part of the blood which, in turn, in a way well understood, absorbs the fluids from the tissues, and, as we would expect the patient rapidly emaciates, etc. It would hardly be expected of the liver, that it would act under these circumstances, for obvious reasons. That bile, therefore, should be absent from the discharges, is not surprising, but a fairly deducible consequence. The same may be said of the kidneys and urine.

From this excessive loss of water, the patient has a raging thirst, but *any* one acquainted with the principles of osmosis, will see it is impossible much water should be absorbed from a surface, or a mucous membrane, from which water is oozing out in streams, as one might not inappropriately say. What has been said about water, may be *also* said of medicines.

When the nervous system recovers from the shock (if I may so speak) experienced, the heart and muscular arteries, and various secreting organs, etc., resume, *pari passu*, their functions. The pulse returns, the portal circulation is restored, the discharges cease, the secretions reäppear, remedies begin to have their appropriate effects, and so on to recovery, many times in a space far too short (from the inception of the disease until recovery) for us to suppose it essentially a *blood disease.* Such, in view of the above conclusions, are my views, of the pathology of cholera.

Now, what shall be the treatment of cholera in the stage of collapse, which, in fully developed cases, is the only one of much importance?

1st. Remedies, administered in the ordinary way, give but feeble promise of effect in this stage, as an appeal to experience will show. The reason is obvious. If the intention is that the remedy shall be absorbed from the *stomach*, and, in the blood be carried through the body, the state of the portal circulation, it is almost needless to say, forbids it. Notwithstanding this, remedies might be employed, such as calomel, or powerful stimulants, such as will arouse the mucous membrane and stimulate to contraction the engorged vessels, and, possibly, indirectly the neighboring nervous centres.

2d. In view of the coldness of the surface, and condition of its capillary circulation, we would employ heat, severe friction, and powerful external stimulants assiduously, to rouse the susceptibility of the skin, and promote the circulation in the *surface* of the body, and for the purpose of rousing, indirectly, the nervous system, through irritation and stimulation of a surface so large, and, ordinarily, so sensitive, I would use the most efficient stimulants and irritants. But, in view of the probable state of the organic nervous system, which calls for the most active and direct stimulation, which can only be effectually made when stimulants are introduced into the blood, and are carried in it to such parts of the organism as require their agency, and in view of the state of the mucous membrane of the stomach, etc., by which it is unfitted for absorption only imperfectly, I say, in view of all this, we may well look for some other channels through which we may introduce such remedies as are required, or *seem* to be. There are two or three ways in which this may be accomplished:—

1st. By extensive faradization of various parts of the body, and by currents sent in a variety of directions through the body.

2d. By inhalations, as of chloroform, ether, bromine, etc.

3d. By hypodermic injections. In this way, we may introduce beneath the skin, in many places, known quantities of various soluble substances, such as morphia, strychnia, quinia, the active principles of coffee and tea, etc., with the certain assurance they will be rapidly absorbed by the flaccid vessels and carried to all parts of the body, and in this way made to act speedily on those parts of the organism upon reaching which, with appropriate agencies, the fate of many cases depends. Such, in brief, are my views of the pathology and treatment of cholera.

To fully unfold or set forth the subject glanced at in this paper, would require a *series* of essays.

The views advocated in this paper have been incidentally referred to in a report, made to the Illinois State Medical Society, on "Cerebro-Spinal Meningitis." They are presented here because they are deemed important, and especially in connexion with our expected visitant—cholera, in which I have briefly tried to apply them; and because they have not seemed to me, for some time past, to have been sufficiently recognized by the profession.

It would be a pleasant task to apply these same principles to the inflammatory process, in all its phases, in exudations and the like, and various pathological states, and see how far, if these principles be admitted, the prevalent doctrines of inflammation are from the truth, etc. But I have neither time nor the space for this.

